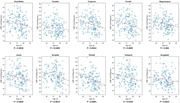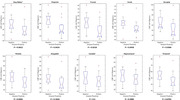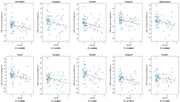# Higher amyloid burden is associated with faster reduction in cerebral blood flow: a longitudinal study

**DOI:** 10.1002/alz.093317

**Published:** 2025-01-03

**Authors:** Tianrui Zhao, Jianing Tang, Jamie Terner, Elizabeth B Joe, Meredith N Braskie, Helena C Chui, Vasilis Marmarelis, Lirong Yan

**Affiliations:** ^1^ Department of Radiology, Northwestern University, Chicago, IL USA; ^2^ Stevens Neuroimaging and Informatics Institute, University of Southern California, Los Angeles, CA USA; ^3^ Department of Neurology, Keck School of Medicine, University of Southern California, Los Angeles, CA USA; ^4^ Stevens Neuroimaging and Informatics Institute, Los Angeles, CA USA; ^5^ Department of Biomedical Engineering, University of Southern California, Los Angeles, CA USA

## Abstract

**Background:**

Amyloid accumulation is one of the main pathophysiological hallmarks of Alzheimer’s disease (AD) and is closely associated with neuronal dysfunction and cognitive decline. Except for amyloid pathology, accumulating evidence has shown vascular dysfunctions, such as reduced cerebral blood flow (CBF), also contribute to AD pathophysiology. However, there remains limited research about the longitudinal changes between amyloid accumulation and CBF. The present study aims to address the gap through conducting both cross‐sectional and longitudinal studies to investigate the relationship between amyloid burden and CBF changes.

**Method:**

168 participants (72.5 ± 8.5 years, 86 female) were enrolled in this study. CBF measurements were obtained from each participant using single‐delay pCASL. Among participants, 94 underwent amyloid PET scans. 72 out of them received a second pCASL MRI scan for one‐year follow‐up. Standardized update value ratio (SUVR) was calculated from PET data. A global SUVR of 1.08 was used to define amyloid positivity. Mean CBF and SUVR were extracted from gray matter and nine AD‐related brain regions.

Linear mixed effects model was employed to examine the association between baseline CBF and baseline SUVR with correcting for age and gender; A paired t‐test assessed CBF difference between the amyloid positive and negative groups; Multivariate linear regression was performed to investigate the association of baseline SUVR level with CBF changes by considering the follow‐up CBF as dependent variable and baseline CBF, SUVR level, time‐gap, age, and gender as independent variables.

**Result:**

Baseline CBF showed significant correlations with age in gray matter and 8 AD‐related regions (Figure 1). Baseline CBF demonstrated a strong association with amyloid positivity in gray matter and 5 AD‐related regions (Figure 2). No significances were observed between baseline CBF values and SUVR levels in all 9 regions. However, baseline SUVR level was significantly associated with longitudinal CBF changes in 8 AD related regions (Figure 3). These results suggest a higher amyloid burden may predict faster CBF reduction during the progression of AD.

**Conclusion:**

Our results demonstrate that longitudinal CBF changes are strongly associated with baseline SUVR levels both globally and regionally, suggesting that increased amyloid burden may affect neurological dysfunction leading to CBF reduction over time.